# Intensity-specific effects of acute exercise on human memory function: considerations for the timing of exercise and the type of memory

**DOI:** 10.15171/hpp.2018.36

**Published:** 2018-10-27

**Authors:** Paul D. Loprinzi

**Affiliations:** Exercise & Memory Laboratory, Department of Health, Exercise Science and Recreation Management, The University of Mississippi, University, MS 38677, USA

**Keywords:** Episodic memory, High-intensity, Moderate-intensity, Walking, Working memory

## Abstract

**Background:** The purpose of this review was to evaluate whether acute exercise intensities have unique effects on memory function, and whether this is influenced by memory type as well as the temporality of the acute exercise bout.

**Methods:** A systematic review was employed, using several databases (PubMed, PsychInfo,Sports Discus, Google Scholar, Embase).

**Results:** In total, 9 articles met the study criteria. All 9 studies evaluated either working memory capacity or episodic-related memory function. The main findings across these studies were 1) when acute exercise occurs before the memory task, high-intensity exercise may be less favorable for working memory but may favor episodic memory; 2) when acute exercise occurs during the memory task, high-intensity exercise may be less favorable for working memory capacity; and 3) high-intensity exercise may not associate with long-term memory function when it occurs shortly after memory encoding.

**Conclusion: **The relationship between acute exercise and memory is complex and may vary based on the intensity of exercise, the temporality of exercise, and the memory type evaluated.

## Introduction

Unquestionably, memory function is critical for optimal daily function. Retrospective memory recall refers to the ability to recall past events, with episodic memory defined as the retrospective recall of past episodes or events in a spatio-temporal context (what-where-when aspects of memory). In addition to episodic memory, another commonly evaluated memory type includes working memory capacity, which refers to the transient repository of information, often while occurring during the concurrent processing of other information (e.g., remembering your hotel room number during the day).

Emerging work suggests that acute exercise (and chronic) may subserve both episodic and working memory capacity.^1-5^ We have previously discussed the potential mechanisms through which exercise may influence episodic memory function.^6,7^ These postulated exercise-related mechanisms include, for example, 1) Enhanced neuronal excitability; 2) Enhanced attentional resource allocation to facilitate memory encoding; 3) Upregulation of AMPA receptor levels, opening NMDA channels, and increasing EPSP (excitatory post-synaptic potentials) in the hippocampus; 4) The priming of neurons to be encoded in the memory trace by increasing CREB transcription; 5) BDNF (brain-derived neurotropic factor) expression; and 6) Enhanced dendritic spine growth. Notably, these exercise-induced changes are likely to occur in brain structures (e.g., prefrontal cortex and hippocampus) that subserve memory function. In the context of working memory, acute exercise may enhance this memory type via modulation of norepinephrine and dopamine levels.^8^ For example, D_1_ receptor stimulation enhances the excitability of prefrontal pyramidal cells and potentiates glutamate gated currents.^8^ Importantly, working memory is optimized at intermediate levels of D_1_ receptor stimulation and is degraded by either too little, or too much activation of this dopamine receptor. Thus, exercise intensity may play a critical role in memory function.

The purpose of this brief review was to evaluate the literature to examine the extent to which acute exercise intensity influences memory function. Although various reviews have discussed the effects of exercise on memory,^3,5,6^ no review, to my knowledge, has specifically evaluated intensity-specific effects of acute exercise on human memory. Some, however, have looked to see if memory outcomes varied based on whether studies compared no exercise to vigorous-intensity exercise, or no exercise to moderate-intensity exercise.^3^ What is lacking in the literature is isolating studies that have made direct comparisons to multiple exercise intensities (i.e., control vs. moderate-intensity vs. high-intensity), which is the focus of the present review. Herein, I also examine whether memory type (e.g., working memory vs. episodic memory) and the temporal effects of exercise (i.e., acute exercise occurring before, during or after the memory task) play an important role in the exercise intensity-memory relationship.

## Materials and Methods

Studies were identified using electronic databases, including PubMed, PsychInfo, Sports Discus, Google Scholar, and Embase.^9^ In alignment with PRISMA guidelines, computerized searches were conducted up until July 10, 2018, identifying articles published prior to this date (no restriction was placed on how far back the study was published). The search terms included: exercise; intensity; cognition; cognitive function; memory; low-intensity; moderate-intensity; and high-intensity (and their combinations). 

To be eligible for inclusion in this systematic review, studies had to:

- Be published in English.
- Be conducted in humans.
- Employ an experimental design.
- Independent variable had to be a measure of acute exercise (not chronic exercise^10-12^). At least 2 different exercise intensity levels (not exercise duration^13,14^) had to be compared (e.g., moderate-intensity vs. high-intensity exercise).
- Include a cognitive-related memory assessment (e.g., episodic memory, working memory). Studies that employed an overall cognitive function assessment that included memory as part of this global score were not included. Additionally, studies that focused on motor memory were excluded.^15-18^
- Not include a stress-induced neurotoxicity paradigm (e.g., looking at the protective effects of exercise intensity on attenuating stress-induced memory impairment)^19-22^ or another type of environmental stimuli (e.g., hypoxia) that could alter memory function.^23^

## Results

The computerized searches revealed 526 unique articles. The title and abstract of each of these articles were reviewed. Among these 526 articles, 9 articles met the study criteria listed above. Table 1 displays the extraction table for the results of the 9 evaluated studies.

Among the 9 evaluated studies,^2,24-31^ all, with the exception of one (adolescents),^26^ were conducted among young adults (18-30 years). All 9 studies evaluated either working memory capacity or episodic-related memory function. Five studies employed a between-subject experimental design, with the other 4 employing a within-subject experimental design. The exercise protocols varied considerably, including a short maximal treadmill bout^25^; 40-minute of low impact running vs. 2 x 3 minute sprints^24^; 12-minutes of running at 50%-65% vs. 70%-85% HR_max_^26^; 30-minutes of treadmill exercise at either 40%-50%, 51%-70%, or 71%-85% HR_max_^27^; 30-minutes of treadmill exercise at Vt (ventilatory threshold) + 20%, Vt – 20%, or VO_2max_ protocol^2^; 30-minutes of cycle exercise at <57 or 80% of HR_max_^28^; 60-minutes of cycle exercise at 90% of Vt vs. 90% of Vt with intermittent sprints^29^; 10-minutes of walking slowly vs. stepping exercise with holding 1kg weight^30^; and 60-minutes of cycle exercise at Vt + 10% vs. <30 W.^31^

In addition to variations in the study design (between-subject vs. within-subject), memory type (working memory vs. episodic memory), and exercise protocol (cycling vs. treadmill; and considerable variations in different intensities), the temporal sequence of the exercise and memory assessments also varied. For example, some studies implemented the exercise protocol prior to the memory assessment,^2,24,26,27,30^ others evaluated memory during exercise,^25,29,31^ and others implemented the exercise bout after memory encoding (i.e., during early memory consolidation).^28^

Regarding the main findings, for the studies implementing the exercise protocol prior to the memory assessment,^2,24,26,27,30^ 4 evaluated episodic memory and one evaluated working memory. In the working memory study,^26^ the initial main analysis did not demonstrate a statistically significant effect, but follow-up analyses demonstrated that the lower exercise-intensity group (50%-65% HR_max_ vs. 70%-85% HR_max_) appeared to have greater increases in working memory, and low baseline performers on the working memory task also demonstrated suggestive evidence of improvements from high-intensity exercise. Among the 4 episodic memory studies, one study did not observe any intensity-specific differences on memory,^27^ whereas the other 3 studies provided evidence that the higher-intensity protocol was advantageous in memory.^2,24,30^ For example, one study^24^ showed that a few short sprints (3-minute) was more effective in enhancing learning when compared to a 40-minute low impact jog, and those with greater exercise-induced increases in epinephrine had greater memory retention at both the 1-week and 6-8 month follow-up periods. Another study^2^ demonstrated that a short-duration, maximal bout of exercise (vs. Vt + 20% and Vt-20%) was the most effective in enhancing long-term (24-hour follow-up) memory. Lastly, one study^30^ showed that 10-minute of stepping exercise with a hand-held weight (vs. slow walking) was more effective in enhancing memory recognition 2-days later. Taken together, among these 5 studies evaluating exercise before the memory task, these findings provide suggestive evidence that lower intensity exercise may be more beneficial for working memory, but higher-intensity exercise may favor episodic memory.

Three studies evaluated memory function during the exercise bout.^25,29,31^ All 3 of these studies evaluated working memory capacity. Over a 60-minute bout of cycle exercise, one study^29^ did not observe any intensity-dependent changes in working memory. However, the other 2 studies^25,31^ demonstrated that working memory function was impaired during a bout of high-intensity exercise when compared to lower-intensity exercise; i.e., during a maximal bout of exercise (vs. before and after)^25^ or cycle exercise at Vt + 10% vs. <30 watts.^31^ Taken together, among these 3 studies evaluating memory function during exercise (or during intermittent breaks^25^), these findings provide suggestive evidence that working memory capacity may be impaired during exercise, particularly for higher-intensity exercise.

Lastly, one study implemented the exercise bout after memory encoding (i.e., during early memory consolidation).^28^ This study evaluated episodic memory. This study showed that those in the control group recalled more words at the 2 follow-up assessment periods (20-minute and 24-hour recall). However, the high-intensity group forgot fewer words across the 2 time points, but this could have been a result of a greater capacity for change in the control group.

## Discussion

The purpose of the present review was to evaluate whether there is an intensity-specific effect of acute exercise on memory function. The main findings of this review are as follows. Few studies have evaluated varying acute exercise intensities on memory function, and thus, it is not possible to provide strong conclusions. Despite this, these findings suggest that: 1) when acute exercise occurs before the memory task, high-intensity exercise may be less favorable for working memory but may favor episodic memory; 2) when acute exercise occurs during the memory task, high-intensity exercise may be less favorable for working memory capacity; and 3) high-intensity exercise may not associate with long-term memory function when it occurs shortly after memory encoding. The narrative that follows will discuss each of these 3 points.

The first observation of this review was that there was some suggestive evidence that when acute exercise occurs before the memory task, high-intensity exercise may be less favorable for working memory but may favor episodic memory. Regarding the timing of exercise, other work (not comparing different intensity levels) demonstrates that acute moderate- and high-intensity exercise before memory encoding is optimal when compared to other temporal periods.^3,4,32-34^ Differential effects of exercise intensity on working memory when compared to episodic memory, have been discussed elsewhere.^35^ Moderate-intensity exercise may favor working memory capacity, in particular. Both light and moderate-intensity acute exercise, but not high-intensity exercise, are associated with increased P3 amplitude in tasks related to information processing and executive function, whereas only moderate-intensity acute exercise is associated with shortened P3 in tasks involving executive function.^36-38^

High-intensity exercise, on the other hand, may increase levels of norepinephrine (NE) and dopamine (DA) in the prefrontal cortex, activating β-adrenoceptors and D_1_-receptors, respectively, ultimately activating cAMP, which may dampen neuronal activity (via cAMP opening of nearby K+ channels, which may weaken the effectiveness of nearby synaptic inputs^39^) in the prefrontal cortex, and potentially impair prefrontal cortex function.^40^ There appears to be an inverted U-shaped relationship between NE/DA and working memory function. High levels of NE activate α1 and β1 receptors, which in turn, facilitate glucocorticoid detrimental actions. Both NE and DA have complimentary roles, with high levels increasing the noise-to-signal ratio; at modest levels, D1 receptor activation increases neuronal firing by decreasing firing to non-preferred inputs (i.e., decreasing noise), whereas NE increases firing to preferred inputs (i.e., increasing signals).^40^ On the other hand, and in contrast to impaired working memory capacity in the prefrontal cortex, elevated levels of catecholamines can enhance synaptic plasticity in the hippocampus.^41,42^ For example, elevated levels of NE can induce various intracellular signaling pathways (e.g., PKA) to facilitate CREB transcription, and in turn, subserve long-term potentiation.^43^ Relatedly, animal work demonstrates that increased running speed is accompanied by systematic increases in the frequency of CA1 network oscillations spanning the gamma frequency range.^44^

Another observation from the evaluated studies of this review was that when acute exercise occurs during the memory task, high-intensity exercise may be less favorable for working memory capacity. This may be explained, in part, from the transient hypofrontality hypothesis,^45,46^ which hypotheses that during exercise there is a relative shift in neural activity away from the prefrontal cortex in order to sustain neural activation in other brain regions that are more critical for movement. Research shows that during exercise, performance on tasks demanding prefrontal-dependent cognition are impaired, while cognitive processes requiring little prefrontal activity are unaffected.^47^ I am not suggesting, however, that episodic memory function may be completely preserved during exercise, as the prefrontal cortex may still play an important role in episodic memory.^48^ Notably, and when compared to exercising prior to memory encoding, experimental work suggests that episodic memory may be slightly worse when memory encoding occurs during exercise.^32,33,49^

The remaining observation from this review was that high-intensity exercise may not associate with long-term memory function when it occurs shortly after memory encoding. Of course, this should be interpreted with caution as only a single study in this review evaluated a potential differential effect of post-exercise intensity on memory function.^28^ This finding aligns with other related research. Although not directly comparing different exercise intensity levels in a single study, other research suggest that exercising shortly after memory encoding is slightly less advantageous (when compared to exercising prior to encoding) for both moderate-intensity^32,34,49-51^ and high-intensity exercise.^33^ In addition to these laboratory findings evaluating exercise very shortly after memory encoding, other work in free-living settings shows that exercising 1-2 hours after memory encoding may also be less advantageous for long-term memory.^52^ Notably, however, emerging work suggests that exercising 4-hours after memory encoding is associated with enhanced long-term memory function.^53^ Future work is needed that examines different temporal periods of exercise post-encoding to evaluate whether there is an optimal time period to which exercise may enhance the consolidation of memories. Additionally, future research would benefit by evaluating the effects of anaerobic sprints on memory performance, as the studies evaluated herein did not impose short-duration (e.g., 60-seconds or less), high-intensity sprints.

In conclusion, this review suggests an intensity-specific effect of exercise on memory and also highlights that results may differ based on memory type and the temporality of memory assessment. Specifically, when acute exercise occurs before the memory task, high-intensity exercise may be less favorable for working memory but may favor episodic memory; when acute exercise occurs during the memory task, high-intensity exercise may be less favorable for working memory capacity; and high-intensity exercise may not associate with long-term memory function when it occurs shortly after memory encoding. Additional work exploring these interrelationships is needed.

## Ethical approval

Not applicable.

## Competing interests

None.

## Funding

None.

## Author’s contribution

PDL conceived the study and wrote the entire manuscript.

**Table 1:** Extraction table of the evaluated studies

**Study**	**Subjects**	**Study Design**	**Exercise Protocol**	**Memory Task**	**Time Period of Assessments**	**Findings**
Winter et al^24^	30 healthy young males (~22 y)	Experimental, between-subject design	Control (15 min), 40-min of low impact running, and 2 sprints of 3-min at increasing speed	Language learning paradigm (episodic memory)	Exercise and then the memory assessment occurred immediately after exercise, 1-week later, and 6-8 months later.	Immediate learning was slightly greater in the high-intensity group. No significant differences for the 1-week follow-up period, but the high-intensity group also had slightly greater memory retention. In the high-intensity exercise group, those with greater increases in epinephrine (vs. below median epinephrine levels) had greater memory retention at both the 1-week and 6-8 month follow-up periods.
Lo Bue-Estes et al^25^	18 youth women (18-25 y)	Experimental, within-subject design	Maximal bout of treadmill running	Arithmetic calculations (working memory)	Working memory assessment occurred pre-exercise, post-exercise, and then at 4 time-points during exercise (25%, 50%, 75%, and 100% of max). The assessments during occurred during a break between transitioning to the faster speed.	Working memory declined during the exercise period but then was enhanced above baseline at the 30-min post-exercise period.
Budde et al^26^	60 healthy high school students (15-16 y)	Experimental, between-subject design	Control, 50-65% HR_max_, and 70-85% HR_max_. Exercise protocol included 12-min of running on a 400-m track	Letter Digit Span Task (working memory)	Memory assessment, exercise, 5-min rest, then memory assessment	No statistically significant group x time interaction. However, the lower exercise-intensity group appeared to have greater increases in working memory. Low baseline performers on the working memory task also demonstrated suggestive evidence of improvements from high-intensity exercise.
Loprinzi and Kane^27^	87 young adults (~21 y)	Experimental, between-subject design	Control, 40%-50% HR_max_, 51-70% HR_max_, or 71%-85% HR_max_. Each bout of exercise lasted 30-min.	Spatial span; paired associates (short-term visuospatial memory)	Exercise and then rested for 15-min (or heart rate was within 10% of resting) before starting the memory task	No statistically significant group x time interaction.
Etnier et al^2^	16 young adults (~23 y)	Experimental, within-subject design	VO_2max_, Vt + 20%, Vt – 20%. The submaximal treadmill exercise bouts lasted 30 min.	RAVLT (episodic memory)	Exercise and then immediately commence memory task	There was no significant group x time interaction effect for learning (RAVLT trials 1-5). Long-term memory (24-h follow-up) was greatest for the maximal exercise condition (VO_2max_).
Hotting et al^28^	81 young healthy adults (18-29 y)	Experimental, between-subject design	Control, <57% HR_max_, ~80% HR_max_. Sessions lasted 30-min. Cycle ergometer exercise.	20-item word list (episodic memory)	Memory encoding, exercise (~10-min after memory encoding), then memory retrieval	Those in the control group recalled more words at the two follow-up assessment periods (20-min and 24-h recall). The high-intensity group forgot fewer words across the two time points, but this could have been a result of a greater room for change in the control group.
Rattray and Smee^29^	20 healthy young adults (~26 y)	Experimental, within-subject design	Control, 90% Vt, 90% of Vt with pick-ups, and 90% of Vt with drop-downs. Exercise (cycle) bout lasted approximately 60-min.	Speed Match task (similar to 1-back condition of n-back task). Measures working memory.	Memory baseline, then exercise, and during the 50th and 55th min during exercise, memory was re-assessed.	No significant condition by time interaction for memory accuracy.
Keyan and Bryant^30^	62 healthy young adults (~21 y)	Experimental, between-subject design	Walking slowly vs. stepping exercise (stepping up and down while hold 1 kg hand weight). Each exercise bout lasted 10-min.	Exposed to images from the IAPS. Memory recall was assessed 2-days later. Memory recognition.	Exercise and then immediately viewed the images	Exercise participants recalled more emotional images than control participants, and more negative than positive images were recalled.
Tempest et al^31^	14 young adults (~23 y)	Experimental, within-subject design	High-intensity exercise (Vt + 10%) and very low-intensity (<30 W). Exercise bouts lasted 60-min and included cycle ergometer exercise	2-back task (working memory).	Exercise, and while exercising, they completed the memory task.	Over the bout of exercise, working memory declined in the high-intensity exercise session, with no change in the low-intensity session.
